# Tetra­kis(pyridazine-κ*N*)bis­(seleno­cyanato-κ*N*)cobalt(II) pyridazine disolvate

**DOI:** 10.1107/S1600536812027742

**Published:** 2012-06-23

**Authors:** Susanne Wöhlert, Mario Wriedt, Inke Jess, Christian Näther

**Affiliations:** a Institut für Anorganische Chemie, Christian-Albrechts-Universität Kiel, Max-Eyth-Strasse 2, 24118 Kiel, Germany; b Department of Chemistry, Texas A&M University, College Station, Texas 77843, USA

## Abstract

Reaction of cobalt(II) nitrate with potassium seleno­cyanate and pyridazine leads to single crystals of the title compound, [Co(NCSe)_2_(C_4_H_4_N_2_)_4_]·2C_4_H_4_N_2_, which is isotypic with its nickel(II) thio­cyanate analogue. The Co^2+^ cations are coordinated by two *N*-bonded seleno­cyanate ligands and four N atoms from four pyridazine ligands into discrete complexes. The complexes are arranged into layers parallel to (001). These layers are separated by additional non-coordinating pyridazine ligands.

## Related literature

For background to this work, including related thio­cyanato compounds, see: Boeckmann & Näther (2010 ▶, 2011 ▶); Wöhlert *et al.* (2011 ▶). For the isotypic Ni thio­cyanate analogue, see: Wöhlert *et al.* (2012 ▶). For related pyridazine coordination compounds, see: Boeckmann *et al.* (2011 ▶); Lloret *et al.* (1998 ▶). For crystallographic analysis, see: Spek (2009 ▶).
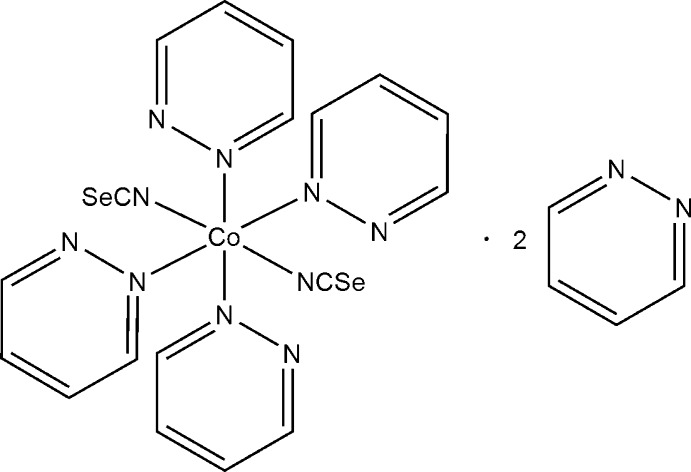


## Experimental

### 

#### Crystal data

[Co(NCSe)_2_(C_4_H_4_N_2_)_4_]·2C_4_H_4_N_2_
*M* *_r_* = 749.44
Triclinic, 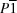

*a* = 11.2138 (9) Å
*b* = 12.0996 (11) Å
*c* = 12.7033 (11) Å
α = 62.206 (9)°
β = 88.827 (10)°
γ = 88.682 (10)°
*V* = 1524.3 (2) Å^3^
*Z* = 2
Mo *K*α radiation
μ = 2.99 mm^−1^
*T* = 170 K
0.15 × 0.11 × 0.08 mm

#### Data collection

STOE IPDS-1 diffractometer
Absorption correction: numerical (*X-SHAPE* and *X-RED32*; Stoe & Cie, 2008 ▶) *T*_min_ = 0.579, *T*_max_ = 0.697
16685 measured reflections
7160 independent reflections
4872 reflections with *I* > 2σ(*I*)
*R*_int_ = 0.047

#### Refinement

*R*[*F* ^2^ > 2σ(*F* ^2^)] = 0.038
*wR*(*F* ^2^) = 0.100
*S* = 0.95
7160 reflections
388 parameters
H-atom parameters constrained
Δρ_max_ = 0.42 e Å^−3^
Δρ_min_ = −0.74 e Å^−3^

### 

Data collection: *X-AREA* (Stoe & Cie, 2008 ▶); cell refinement: *X-AREA*; data reduction: *X-AREA*; program(s) used to solve structure: *SHELXS97* (Sheldrick, 2008 ▶); program(s) used to refine structure: *SHELXL97* (Sheldrick, 2008 ▶); molecular graphics: *XP* in *SHELXTL* (Sheldrick, 2008 ▶) and *DIAMOND* (Brandenburg, 2011 ▶); software used to prepare material for publication: *XCIF* in *SHELXTL*.

## Supplementary Material

Crystal structure: contains datablock(s) I, global. DOI: 10.1107/S1600536812027742/wm2650sup1.cif

Structure factors: contains datablock(s) I. DOI: 10.1107/S1600536812027742/wm2650Isup2.hkl

Additional supplementary materials:  crystallographic information; 3D view; checkCIF report

## Figures and Tables

**Table 1:** Selected bond lengths (Å)

Co1—N1	2.084 (2)
Co1—N2	2.091 (2)
Co1—N20	2.174 (2)
Co1—N40	2.175 (2)
Co1—N10	2.197 (2)
Co1—N30	2.204 (2)

**Fig. 1.**
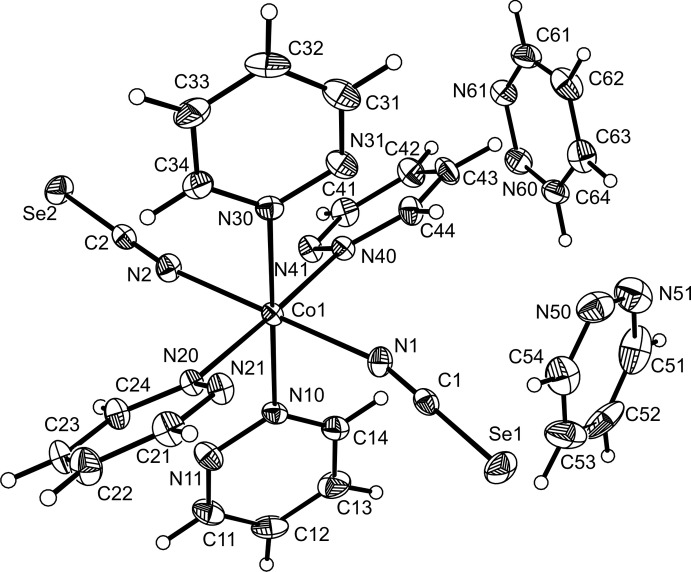
Crystal structure of the title compound with atom labelling and displacement ellipsoids drawn at the 50% probability level.

**Fig. 2.**
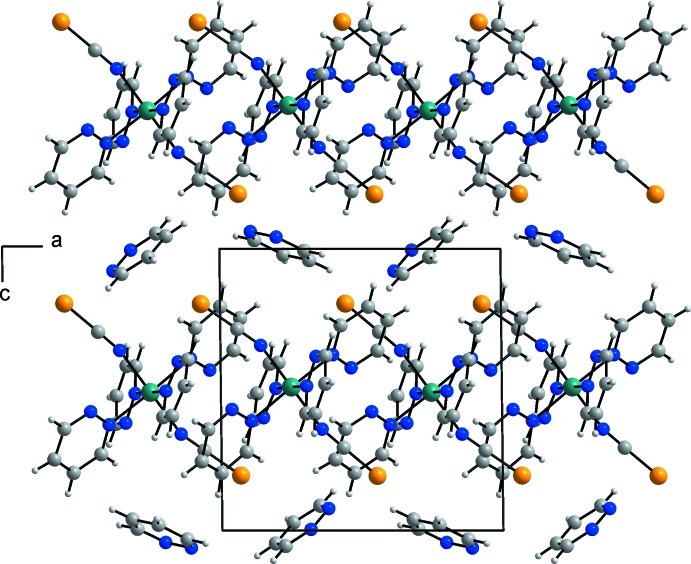
Crystal structure of the title compound in a view along [010].

## supplementary crystallographic information

### Comment

Recently, we have reported on the synthesis and characterization of coordination polymers based on transition metal(II) thiocyanates and different monodentate and bidentate co-ligands (Boeckmann & Näther, 2010, 2011; Wöhlert *et al.*, 2011). In the course of these investigations we have found that the thiocyanato compounds are frequently isotypic with their selenocyanato analogues and exhibit a similar thermal reactivity and a similar magnetic behaviour (Boeckmann & Näther, 2011). In view of these results, we tried to prepare similar compounds based on pyridazine as co-ligand which results in the formation of single-crystals of the title compound, which are isotypic to [Ni(NCS)_2_(N_2_C_4_H_4_)_4_]^.^2(N_2_C_4_H_4_) reported recently (Wöhlert *et al.*, 2012).

In the crystal structure each cobalt(II) cation is coordinated by two terminal N-bonded selenocyanate anions and four pyridazine ligands into discrete complexes (Fig. 1). The octahedral coordination of the cobalt(II) cations is slightly distorted with distances in the range of 2.084 (2) to 2.204 (2) Å and angles ranging from 87.31 (9) ° to 179.70 (10) °. The discrete complexes are arranged into layers parallel to (001) (Fig. 2). These layers are separated by additional non-coordinating pyridazine ligands. The shortest intermolecular Co···Co distances amount to 8.2074 (11) Å.

It must be noted that similar discrete complexes based on cobalt and cadmium as counter cations are already reported in literature (Boeckmann *et al.*, 2011; Lloret *et al.*, 1998).

### Experimental

Cobalt(II) nitrate hexahydrate (Co(NO_3_)_2_^.^6H_2_O) and potassium selenocyanate (KNCSe) as well as pyridazine were obtained from Alfa Aesar. All chemicals were used without further purification. 0.125 mmol (36.4 mg) Co(NO_3_)_2_^.^6H_2_O and 0.25 mmol (36.0 mg) KNCSe were reacted in 2.76 mmol (200 µ*L*) pyridazine. Orange single-crystals of the title compound were obtained after three days.

### Refinement

All H atoms could be located in difference maps but were positioned with idealized geometry and were refined isotropic with *U*_iso_(H) = 1.2 *U*_eq_(C) of the parent atom using a riding model with C—H = 0.95 Å. *PLATON* (Spek, 2009) detect a pseudo-translation which is without any relevance because our investigations cleary shows that the symmetry and the space group is correct.

### Crystal data

**Table:**

[Co(SeCN)_2_(C_4_H_4_N_2_)_4_]·2C_4_H_4_N_2_	*Z* = 2
*M**_r_* = 749.44	*F*(000) = 746
Triclinic, *P*1	*D*_x_ = 1.633 Mg m^−^^3^
Hall symbol: -P 1	Mo *K*α radiation, λ = 0.71073 Å
*a* = 11.2138 (9) Å	Cell parameters from 16685 reflections
*b* = 12.0996 (11) Å	θ = 2.6–28.0°
*c* = 12.7033 (11) Å	µ = 2.99 mm^−^^1^
α = 62.206 (9)°	*T* = 170 K
β = 88.827 (10)°	Block, orange
γ = 88.682 (10)°	0.15 × 0.11 × 0.08 mm
*V* = 1524.3 (2) Å^3^	

### Data collection

**Table:**

STOE IPDS-1 diffractometer	7160 independent reflections
Radiation source: fine-focus sealed tube	4872 reflections with *I* > 2σ(*I*)
Graphite monochromator	*R*_int_ = 0.047
phi scan	θ_max_ = 28.0°, θ_min_ = 2.6°
Absorption correction: numerical (*X-SHAPE* and *X-RED32*; Stoe & Cie, 2008)	*h* = −14→14
*T*_min_ = 0.579, *T*_max_ = 0.697	*k* = −15→15
16685 measured reflections	*l* = −16→16

### Refinement

**Table:**

Refinement on *F*^2^	Primary atom site location: structure-invariant direct methods
Least-squares matrix: full	Secondary atom site location: difference Fourier map
*R*[*F*^2^ > 2σ(*F*^2^)] = 0.038	Hydrogen site location: inferred from neighbouring sites
*wR*(*F*^2^) = 0.100	H-atom parameters constrained
*S* = 0.95	*w* = 1/[σ^2^(*F*_o_^2^) + (0.0561*P*)^2^] where *P* = (*F*_o_^2^ + 2*F*_c_^2^)/3
7160 reflections	(Δ/σ)_max_ = 0.001
388 parameters	Δρ_max_ = 0.42 e Å^−^^3^
0 restraints	Δρ_min_ = −0.74 e Å^−^^3^

### Special details

**Table:**

Geometry. All e.s.d.'s (except the e.s.d. in the dihedral angle between two l.s. planes) are estimated using the full covariance matrix. The cell e.s.d.'s are taken into account individually in the estimation of e.s.d.'s in distances, angles and torsion angles; correlations between e.s.d.'s in cell parameters are only used when they are defined by crystal symmetry. An approximate (isotropic) treatment of cell e.s.d.'s is used for estimating e.s.d.'s involving l.s. planes.
Refinement. Refinement of *F*^2^ against ALL reflections. The weighted *R*-factor *wR* and goodness of fit *S* are based on *F*^2^, conventional *R*-factors *R* are based on *F*, with *F* set to zero for negative *F*^2^. The threshold expression of *F*^2^ > σ(*F*^2^) is used only for calculating *R*-factors(gt) *etc*. and is not relevant to the choice of reflections for refinement. *R*-factors based on *F*^2^ are statistically about twice as large as those based on *F*, and *R*- factors based on ALL data will be even larger.

### Fractional atomic coordinates and isotropic or equivalent isotropic displacement parameters (Å^2^)

**Table:**

	*x*	*y*	*z*	*U*_iso_*/*U*_eq_	
Co1	0.74918 (3)	0.74770 (4)	0.50943 (3)	0.01251 (9)	
N1	0.8568 (2)	0.6511 (2)	0.6576 (2)	0.0193 (5)	
C1	0.9395 (3)	0.6035 (3)	0.7155 (3)	0.0170 (6)	
Se1	1.06840 (3)	0.53113 (3)	0.80450 (3)	0.02804 (10)	
N2	0.6414 (2)	0.8434 (2)	0.3603 (2)	0.0194 (5)	
C2	0.5651 (3)	0.8969 (3)	0.2944 (3)	0.0166 (6)	
Se2	0.44604 (3)	0.97836 (3)	0.19407 (3)	0.02555 (10)	
N10	0.8043 (2)	0.9230 (2)	0.5048 (2)	0.0166 (5)	
N11	0.8491 (2)	1.0109 (2)	0.4008 (2)	0.0224 (5)	
C11	0.8829 (3)	1.1191 (3)	0.3937 (3)	0.0253 (7)	
H11	0.9165	1.1796	0.3208	0.030*	
C12	0.8715 (3)	1.1488 (3)	0.4871 (3)	0.0282 (7)	
H12	0.8946	1.2277	0.4782	0.034*	
C13	0.8255 (3)	1.0588 (3)	0.5925 (3)	0.0307 (8)	
H13	0.8157	1.0728	0.6598	0.037*	
C14	0.7938 (3)	0.9459 (3)	0.5969 (3)	0.0236 (7)	
H14	0.7630	0.8821	0.6697	0.028*	
N20	0.8926 (2)	0.7545 (2)	0.3889 (2)	0.0140 (5)	
N21	0.9612 (2)	0.6494 (2)	0.4300 (2)	0.0169 (5)	
C21	1.0463 (3)	0.6422 (3)	0.3595 (3)	0.0219 (6)	
H21	1.0933	0.5677	0.3882	0.026*	
C22	1.0709 (3)	0.7375 (3)	0.2457 (3)	0.0273 (7)	
H22	1.1337	0.7294	0.1983	0.033*	
C23	1.0007 (3)	0.8436 (3)	0.2047 (3)	0.0223 (6)	
H23	1.0133	0.9119	0.1279	0.027*	
C24	0.9105 (3)	0.8474 (3)	0.2800 (3)	0.0189 (6)	
H24	0.8597	0.9192	0.2525	0.023*	
N30	0.6947 (2)	0.5719 (2)	0.5135 (2)	0.0170 (5)	
N31	0.6603 (2)	0.4772 (2)	0.6183 (2)	0.0223 (5)	
C31	0.6270 (3)	0.3707 (3)	0.6204 (3)	0.0290 (7)	
H31	0.6007	0.3049	0.6941	0.035*	
C32	0.6284 (3)	0.3506 (3)	0.5212 (3)	0.0321 (8)	
H32	0.6048	0.2732	0.5266	0.038*	
C33	0.6653 (3)	0.4469 (4)	0.4155 (3)	0.0325 (8)	
H33	0.6692	0.4388	0.3446	0.039*	
C34	0.6971 (3)	0.5576 (3)	0.4160 (3)	0.0240 (7)	
H34	0.7216	0.6260	0.3432	0.029*	
N40	0.6010 (2)	0.7439 (2)	0.6242 (2)	0.0151 (5)	
N41	0.5359 (2)	0.8514 (2)	0.5780 (2)	0.0180 (5)	
C41	0.4414 (3)	0.8588 (3)	0.6389 (3)	0.0220 (6)	
H41	0.3961	0.9345	0.6065	0.026*	
C42	0.4049 (3)	0.7623 (3)	0.7473 (3)	0.0249 (7)	
H42	0.3354	0.7707	0.7872	0.030*	
C43	0.4725 (3)	0.6542 (3)	0.7949 (3)	0.0228 (7)	
H43	0.4525	0.5856	0.8694	0.027*	
C44	0.5718 (3)	0.6497 (3)	0.7289 (3)	0.0199 (6)	
H44	0.6205	0.5763	0.7602	0.024*	
N50	0.6844 (3)	0.6254 (3)	1.0041 (3)	0.0348 (7)	
N51	0.6207 (3)	0.6544 (3)	1.0784 (3)	0.0413 (8)	
C51	0.6508 (4)	0.7539 (4)	1.0884 (4)	0.0467 (11)	
H51	0.6039	0.7751	1.1396	0.056*	
C52	0.7464 (4)	0.8295 (4)	1.0291 (4)	0.0470 (12)	
H52	0.7661	0.8992	1.0404	0.056*	
C53	0.8102 (3)	0.7995 (4)	0.9544 (4)	0.0456 (11)	
H53	0.8763	0.8477	0.9100	0.055*	
C54	0.7752 (3)	0.6955 (4)	0.9454 (4)	0.0373 (9)	
H54	0.8195	0.6732	0.8935	0.045*	
N60	0.2024 (3)	0.8989 (3)	0.9578 (3)	0.0301 (6)	
N61	0.1110 (3)	0.8398 (3)	0.9384 (3)	0.0300 (6)	
C61	0.1245 (3)	0.7193 (3)	0.9688 (3)	0.0320 (8)	
H61	0.0601	0.6787	0.9546	0.038*	
C62	0.2272 (3)	0.6484 (3)	1.0205 (3)	0.0325 (8)	
H62	0.2328	0.5622	1.0412	0.039*	
C63	0.3189 (3)	0.7088 (3)	1.0397 (3)	0.0308 (8)	
H63	0.3913	0.6663	1.0745	0.037*	
C64	0.3023 (3)	0.8353 (3)	1.0065 (3)	0.0281 (7)	
H64	0.3656	0.8784	1.0191	0.034*	

### Atomic displacement parameters (Å^2^)

**Table:**

	*U*^11^	*U*^22^	*U*^33^	*U*^12^	*U*^13^	*U*^23^
Co1	0.01260 (17)	0.00907 (17)	0.01339 (18)	0.00259 (13)	−0.00004 (13)	−0.00327 (14)
N1	0.0210 (12)	0.0180 (13)	0.0168 (12)	0.0044 (10)	−0.0027 (10)	−0.0064 (11)
C1	0.0224 (14)	0.0111 (14)	0.0165 (14)	−0.0024 (12)	0.0025 (11)	−0.0056 (11)
Se1	0.02541 (17)	0.02162 (19)	0.02991 (18)	0.00417 (14)	−0.01359 (14)	−0.00566 (15)
N2	0.0195 (12)	0.0165 (13)	0.0185 (12)	0.0046 (10)	−0.0032 (10)	−0.0051 (11)
C2	0.0190 (14)	0.0130 (14)	0.0174 (14)	−0.0002 (11)	0.0033 (11)	−0.0069 (12)
Se2	0.02245 (16)	0.02343 (19)	0.02468 (17)	0.00506 (13)	−0.01067 (13)	−0.00593 (14)
N10	0.0152 (11)	0.0115 (12)	0.0198 (12)	−0.0001 (9)	−0.0010 (9)	−0.0046 (10)
N11	0.0274 (13)	0.0140 (13)	0.0243 (13)	−0.0024 (11)	0.0058 (11)	−0.0078 (11)
C11	0.0258 (15)	0.0143 (15)	0.0315 (17)	−0.0040 (13)	0.0030 (13)	−0.0071 (14)
C12	0.0231 (15)	0.0214 (17)	0.045 (2)	−0.0046 (13)	−0.0019 (14)	−0.0196 (16)
C13	0.0326 (18)	0.037 (2)	0.0356 (19)	−0.0078 (16)	0.0009 (15)	−0.0273 (18)
C14	0.0239 (15)	0.0245 (17)	0.0237 (16)	−0.0069 (13)	0.0017 (12)	−0.0121 (14)
N20	0.0146 (11)	0.0123 (12)	0.0145 (11)	0.0027 (9)	0.0014 (9)	−0.0060 (10)
N21	0.0187 (11)	0.0129 (12)	0.0168 (12)	0.0063 (10)	−0.0003 (9)	−0.0052 (10)
C21	0.0210 (14)	0.0201 (16)	0.0254 (16)	0.0068 (13)	0.0021 (12)	−0.0117 (14)
C22	0.0249 (16)	0.033 (2)	0.0251 (16)	0.0050 (14)	0.0051 (13)	−0.0145 (15)
C23	0.0274 (16)	0.0206 (16)	0.0140 (14)	−0.0009 (13)	0.0042 (12)	−0.0041 (12)
C24	0.0234 (14)	0.0144 (15)	0.0166 (14)	0.0031 (12)	0.0011 (11)	−0.0054 (12)
N30	0.0156 (11)	0.0142 (12)	0.0210 (12)	0.0002 (10)	−0.0011 (9)	−0.0081 (10)
N31	0.0235 (13)	0.0161 (13)	0.0256 (13)	−0.0021 (11)	0.0038 (11)	−0.0083 (11)
C31	0.0313 (17)	0.0179 (16)	0.0352 (18)	−0.0046 (14)	0.0055 (14)	−0.0102 (15)
C32	0.0259 (17)	0.0266 (19)	0.050 (2)	−0.0100 (15)	0.0035 (16)	−0.0226 (18)
C33	0.0369 (19)	0.037 (2)	0.0339 (19)	−0.0111 (17)	−0.0009 (15)	−0.0251 (18)
C34	0.0244 (15)	0.0237 (17)	0.0238 (16)	−0.0037 (13)	−0.0027 (13)	−0.0108 (14)
N40	0.0142 (11)	0.0125 (12)	0.0165 (11)	0.0028 (9)	−0.0004 (9)	−0.0051 (10)
N41	0.0169 (11)	0.0154 (13)	0.0183 (12)	0.0049 (10)	0.0013 (9)	−0.0053 (10)
C41	0.0198 (14)	0.0210 (16)	0.0257 (16)	0.0067 (12)	0.0006 (12)	−0.0117 (14)
C42	0.0226 (15)	0.0308 (18)	0.0232 (16)	−0.0004 (13)	0.0091 (12)	−0.0145 (14)
C43	0.0274 (16)	0.0217 (17)	0.0166 (14)	−0.0065 (13)	0.0079 (12)	−0.0066 (13)
C44	0.0254 (15)	0.0126 (14)	0.0183 (14)	0.0031 (12)	−0.0018 (12)	−0.0042 (12)
N50	0.0330 (16)	0.0267 (16)	0.0481 (19)	0.0020 (13)	−0.0082 (14)	−0.0201 (15)
N51	0.0368 (17)	0.0308 (18)	0.049 (2)	0.0033 (14)	0.0010 (15)	−0.0126 (16)
C51	0.063 (3)	0.042 (2)	0.038 (2)	0.026 (2)	−0.013 (2)	−0.022 (2)
C52	0.059 (3)	0.0233 (19)	0.068 (3)	0.0145 (19)	−0.045 (2)	−0.028 (2)
C53	0.0251 (18)	0.026 (2)	0.064 (3)	−0.0011 (16)	−0.0099 (18)	−0.003 (2)
C54	0.039 (2)	0.033 (2)	0.037 (2)	0.0072 (17)	−0.0001 (16)	−0.0142 (18)
N60	0.0389 (16)	0.0172 (14)	0.0306 (15)	−0.0035 (12)	−0.0028 (13)	−0.0078 (12)
N61	0.0323 (15)	0.0254 (16)	0.0284 (15)	−0.0049 (13)	−0.0009 (12)	−0.0090 (13)
C61	0.0370 (19)	0.0288 (19)	0.0330 (19)	−0.0126 (16)	0.0046 (15)	−0.0164 (16)
C62	0.044 (2)	0.0183 (17)	0.0356 (19)	−0.0008 (15)	0.0095 (16)	−0.0133 (15)
C63	0.0279 (17)	0.033 (2)	0.0293 (18)	0.0060 (15)	0.0038 (14)	−0.0127 (16)
C64	0.0307 (17)	0.0275 (18)	0.0270 (17)	−0.0102 (14)	0.0052 (13)	−0.0132 (15)

### Geometric parameters (Å, º)

**Table:**

Co1—N1	2.084 (2)	C32—C33	1.369 (5)
Co1—N2	2.091 (2)	C32—H32	0.9500
Co1—N20	2.174 (2)	C33—C34	1.398 (5)
Co1—N40	2.175 (2)	C33—H33	0.9500
Co1—N10	2.197 (2)	C34—H34	0.9500
Co1—N30	2.204 (2)	N40—C44	1.327 (4)
N1—C1	1.157 (4)	N40—N41	1.353 (3)
C1—Se1	1.795 (3)	N41—C41	1.325 (4)
N2—C2	1.163 (4)	C41—C42	1.388 (5)
C2—Se2	1.793 (3)	C41—H41	0.9500
N10—C14	1.325 (4)	C42—C43	1.373 (5)
N10—N11	1.348 (4)	C42—H42	0.9500
N11—C11	1.334 (4)	C43—C44	1.395 (4)
C11—C12	1.393 (5)	C43—H43	0.9500
C11—H11	0.9500	C44—H44	0.9500
C12—C13	1.372 (5)	N50—C54	1.316 (5)
C12—H12	0.9500	N50—N51	1.340 (5)
C13—C14	1.395 (5)	N51—C51	1.320 (5)
C13—H13	0.9500	C51—C52	1.387 (7)
C14—H14	0.9500	C51—H51	0.9500
N20—C24	1.329 (4)	C52—C53	1.352 (7)
N20—N21	1.354 (3)	C52—H52	0.9500
N21—C21	1.325 (4)	C53—C54	1.381 (6)
C21—C22	1.392 (5)	C53—H53	0.9500
C21—H21	0.9500	C54—H54	0.9500
C22—C23	1.373 (5)	N60—C64	1.332 (4)
C22—H22	0.9500	N60—N61	1.353 (4)
C23—C24	1.393 (4)	N61—C61	1.328 (5)
C23—H23	0.9500	C61—C62	1.397 (5)
C24—H24	0.9500	C61—H61	0.9500
N30—C34	1.327 (4)	C62—C63	1.365 (5)
N30—N31	1.345 (4)	C62—H62	0.9500
N31—C31	1.337 (4)	C63—C64	1.393 (5)
C31—C32	1.390 (5)	C63—H63	0.9500
C31—H31	0.9500	C64—H64	0.9500
			
N1—Co1—N2	179.50 (11)	N31—C31—H31	118.0
N1—Co1—N20	91.87 (9)	C32—C31—H31	118.0
N2—Co1—N20	87.88 (9)	C33—C32—C31	117.2 (3)
N1—Co1—N40	90.35 (10)	C33—C32—H32	121.4
N2—Co1—N40	89.90 (9)	C31—C32—H32	121.4
N20—Co1—N40	177.76 (8)	C32—C33—C34	117.3 (3)
N1—Co1—N10	88.58 (9)	C32—C33—H33	121.4
N2—Co1—N10	91.87 (9)	C34—C33—H33	121.4
N20—Co1—N10	92.39 (9)	N30—C34—C33	123.1 (3)
N40—Co1—N10	87.93 (9)	N30—C34—H34	118.4
N1—Co1—N30	91.41 (9)	C33—C34—H34	118.4
N2—Co1—N30	88.14 (9)	C44—N40—N41	120.5 (2)
N20—Co1—N30	87.31 (9)	C44—N40—Co1	126.6 (2)
N40—Co1—N30	92.36 (9)	N41—N40—Co1	112.88 (17)
N10—Co1—N30	179.70 (10)	C41—N41—N40	118.2 (3)
C1—N1—Co1	161.1 (2)	N41—C41—C42	123.8 (3)
N1—C1—Se1	179.5 (3)	N41—C41—H41	118.1
C2—N2—Co1	166.1 (3)	C42—C41—H41	118.1
N2—C2—Se2	179.3 (3)	C43—C42—C41	117.8 (3)
C14—N10—N11	119.7 (3)	C43—C42—H42	121.1
C14—N10—Co1	123.5 (2)	C41—C42—H42	121.1
N11—N10—Co1	116.70 (18)	C42—C43—C44	117.0 (3)
C11—N11—N10	118.7 (3)	C42—C43—H43	121.5
N11—C11—C12	123.8 (3)	C44—C43—H43	121.5
N11—C11—H11	118.1	N40—C44—C43	122.6 (3)
C12—C11—H11	118.1	N40—C44—H44	118.7
C13—C12—C11	117.0 (3)	C43—C44—H44	118.7
C13—C12—H12	121.5	C54—N50—N51	119.1 (3)
C11—C12—H12	121.5	C51—N51—N50	118.3 (4)
C12—C13—C14	117.4 (3)	N51—C51—C52	124.4 (4)
C12—C13—H13	121.3	N51—C51—H51	117.8
C14—C13—H13	121.3	C52—C51—H51	117.8
N10—C14—C13	123.3 (3)	C53—C52—C51	116.8 (3)
N10—C14—H14	118.3	C53—C52—H52	121.6
C13—C14—H14	118.3	C51—C52—H52	121.6
C24—N20—N21	120.4 (2)	C52—C53—C54	117.0 (4)
C24—N20—Co1	125.23 (19)	C52—C53—H53	121.5
N21—N20—Co1	114.20 (17)	C54—C53—H53	121.5
C21—N21—N20	118.4 (2)	N50—C54—C53	124.3 (4)
N21—C21—C22	123.8 (3)	N50—C54—H54	117.8
N21—C21—H21	118.1	C53—C54—H54	117.8
C22—C21—H21	118.1	C64—N60—N61	119.5 (3)
C23—C22—C21	117.3 (3)	C61—N61—N60	118.4 (3)
C23—C22—H22	121.3	N61—C61—C62	124.4 (3)
C21—C22—H22	121.3	N61—C61—H61	117.8
C22—C23—C24	117.5 (3)	C62—C61—H61	117.8
C22—C23—H23	121.2	C63—C62—C61	116.8 (3)
C24—C23—H23	121.2	C63—C62—H62	121.6
N20—C24—C23	122.5 (3)	C61—C62—H62	121.6
N20—C24—H24	118.7	C62—C63—C64	117.5 (3)
C23—C24—H24	118.7	C62—C63—H63	121.3
C34—N30—N31	120.1 (3)	C64—C63—H63	121.3
C34—N30—Co1	121.5 (2)	N60—C64—C63	123.5 (3)
N31—N30—Co1	118.45 (19)	N60—C64—H64	118.3
C31—N31—N30	118.3 (3)	C63—C64—H64	118.3
N31—C31—C32	124.0 (3)		
